# Quantifying Online News Media Coverage of the COVID-19 Pandemic: Text Mining Study and Resource

**DOI:** 10.2196/28253

**Published:** 2021-06-02

**Authors:** Konrad Krawczyk, Tadeusz Chelkowski, Daniel J Laydon, Swapnil Mishra, Denise Xifara, Benjamin Gibert, Seth Flaxman, Thomas Mellan, Veit Schwämmle, Richard Röttger, Johannes T Hadsund, Samir Bhatt

**Affiliations:** 1 Department of Mathematics and Computer Science University of Southern Denmark Odense Denmark; 2 Department of Management in the Network Society Kozminski University Warsaw Poland; 3 Department of Infectious Disease Epidemiology MRC Centre for Global Infectious Disease Analysis Imperial College London London United Kingdom; 4 Nupinion London United Kingdom; 5 Department of Mathematics Imperial College London London United Kingdom; 6 Department of Biochemistry and Molecular Biology University of Southern Denmark Odense Denmark; 7 Section of Epidemiology Department of Public Health University of Copenhagen Copenhagen Denmark

**Keywords:** text mining, COVID-19, infoveillance, sentiment analysis, public health

## Abstract

**Background:**

Before the advent of an effective vaccine, nonpharmaceutical interventions, such as mask-wearing, social distancing, and lockdowns, have been the primary measures to combat the COVID-19 pandemic. Such measures are highly effective when there is high population-wide adherence, which requires information on current risks posed by the pandemic alongside a clear exposition of the rules and guidelines in place.

**Objective:**

Here we analyzed online news media coverage of COVID-19. We quantified the total volume of COVID-19 articles, their sentiment polarization, and leading subtopics to act as a reference to inform future communication strategies.

**Methods:**

We collected 26 million news articles from the front pages of 172 major online news sources in 11 countries (available online at SciRide). Using topic detection, we identified COVID-19–related content to quantify the proportion of total coverage the pandemic received in 2020. The sentiment analysis tool Vader was employed to stratify the emotional polarity of COVID-19 reporting. Further topic detection and sentiment analysis was performed on COVID-19 coverage to reveal the leading themes in pandemic reporting and their respective emotional polarizations.

**Results:**

We found that COVID-19 coverage accounted for approximately 25.3% of all front-page online news articles between January and October 2020. Sentiment analysis of English-language sources revealed that overall COVID-19 coverage was not exclusively negatively polarized, suggesting wide heterogeneous reporting of the pandemic. Within this heterogenous coverage, 16% of COVID-19 news articles (or 4% of all English-language articles) can be classified as highly negatively polarized, citing issues such as death, fear, or crisis.

**Conclusions:**

The goal of COVID-19 public health communication is to increase understanding of distancing rules and to maximize the impact of governmental policy. The extent to which the quantity and quality of information from different communication channels (eg, social media, government pages, and news) influence public understanding of public health measures remains to be established. Here we conclude that a quarter of all reporting in 2020 covered COVID-19, which is indicative of information overload. In this capacity, our data and analysis form a quantitative basis for informing health communication strategies along traditional news media channels to minimize the risks of COVID-19 while vaccination is rolled out.

## Introduction

The emergence of the novel coronavirus SARS-CoV-2 and the resultant disease COVID-19 has resulted in an estimated 2.4 million deaths [1,2]. Due to the initial lack of pharmaceutical measures targeting COVID-19, many governments resorted to nonpharmaceutical interventions (NPIs) to control the spread of the pandemic [3,4]. The introduction of NPIs, such as social distancing, mask-wearing, or so-called *lockdowns*, significantly reduced SARS-CoV-2 transmission [5-8]. Therefore, in the absence of effective treatments or widespread rollout of vaccines, NPIs remain an important tool in COVID-19 control [9].

The effectiveness of NPIs is dependent on population-wide adherence to government mandates (eg, social distancing rules, stay-at-home orders, and mask-wearing). Adherence, in turn, is dependent on society’s perception of such measures [9,10], which are shaped by print and digital media. Since adherence to NPIs is linked to public understanding of the guidelines, it is crucial for news sources shaping such knowledge to effectively expound the rules to maximize public response. As evidence accumulates, it is expected that guidelines will shift and be clarified. In the digital age, one of the primary information sources for society is online news [11,12]. Effective communication on the current state of the pandemic and prevention guidelines affects how society adheres to and responds to NPIs and, therefore, influences the severity of the pandemic.

News articles have been previously shown to be an effective way of tracking disease outbreaks, with services such as HealthMap contributing to detecting and tracking disease outbreaks [13,14]. Even if only by virtue of its unprecedented scale, news media has been particularly important in the COVID-19 pandemic [15]. There have been an estimated 38 million English-language articles on COVID-19 [16]. It has further been demonstrated that misinformation on COVID-19 has been widespread and influential, both on social media [17] and in traditional news sources [16]. This can lead to an overload of information that hinders societal response to the pandemic [18].

A particular difficulty of the pandemic is its emotional toll, both from the disease itself and from social distancing measures [19-21]. Emotional toll can be investigated quantitatively by sentiment analysis, which calculates emotional polarization of text from negative, through neutral, to positive. Three previous studies have attempted to quantify emotional toll using sentiment analysis of social media conversations on COVID-19 [22-24]. Counterintuitively, given pandemic subject matter, all three studies of sentiments from COVID-19 conversations on social media showed a higher proportion of positive rather than negative emotions. In contrast, analysis of COVID-19 headlines from 25 English news media sources indicated that 52% of them evoked negative emotions, 30% evoked positive emotions, and 18% evoked neutral emotions [25]. News media can shape behavior toward the pandemic and adherence to control measures. Therefore, extensive negative coverage or contradictory information (ie, information overload) could have detrimental effects to both the mental health of individuals and how effectively society responds to control measures [26].

An earlier study on COVID-19 news coverage by Evanega et al [16] sourced 38 million English-language COVID-19 articles by keyword search from LexisNexis, which indexes 7 million sources. Quantifying the extent of COVID-19 information requires normalizing the absolute number of articles with respect to the number of contributing sources. Likewise, sentiment of COVID-19 coverage also needs to be analyzed in the context of overall negativity of consumed information. Aslam et al [25] analyzed 141,208 COVID-19 headlines, showing that 52% carried negative sentiment. Similar results were reported by Chakraborty and Bose, who collected COVID-19 news articles from GDELT (Global Database of Events, Language, and Tone) and found that pandemic coverage was mostly associated with negative sentiment polarization [27]. Though informative, these studies did not contrast COVID-19 sentiment distribution with the sentiment distribution of the sources they originated from. By contextualizing the sentiment distribution of pandemic reporting within that of the overall coverage, it is possible to draw meaningful conclusions on whether the amount of COVID-19 information is indeed more negatively polarized than what news media consumers are exposed to.

To address the above issues, we collected over 26 million articles from the front pages of 172 major online news sources from 11 countries and compiled these into a reusable database available at SciRide [28]. Firstly, we investigated trends in COVID-19 news with respect to all articles that appeared on the front pages in 2020. Secondly, we analyzed whether articles on COVID-19 were more sentiment-polarized compared with other articles. Finally, we analyzed the leading subtopics in COVID-19 coverage and assessed their sentiment polarization. Overall, our work aimed to elucidate the volume and content of news coverage of COVID-19 in traditional media as a basis for data-driven discussion regarding policy communication in the pandemic.

## Methods

### Curation of a Database of Front-Page News Articles

To assess coverage of COVID-19 and the sentiment it evoked, we analyzed the landing pages from major online news sources in countries with robust media presence. We selected the major online news sources from 11 countries: the United States, the United Kingdom, Canada, Australia, New Zealand, Ireland, Germany, France, Italy, Spain, and Russia. We included an additional *international* category to better reflect the global focus of certain online news sources.

For each country, the major online news sources were identified by reference to profiles in BBC Media, which is an authority in curating global news source information and lists of news sites with the most traffic and is curated by SimilarWeb. Focusing on major national online news sources, as defined by online visibility, captures some of the main sources in shaping societal knowledge and opinions [29]. It should be noted that the focus on online news sources excludes the impact of social media, epistemic communities, and other influences on public perception. However, due to their depth of penetration, political heterogeneity, and reliability, major online news sources provide an excellent proxy of overall public perception.

For each online news source, we collected the archived front-page snapshots dating back to 2015 via Internet Archive [30], cutting off coverage in 2020 at October 15. We used the pre-2020 articles to fine-tune the accuracy of article collection and provide statistics on reporting of certain topics pre–COVID-19. Each front page was sourced for potential news items using a custom-based pipeline we developed (Section 1 in Multimedia Appendix 1). We defined each article as the combination of the metadata elements title and description—sometimes referred to as headline and subheading—that are broadly akin to titles and abstracts in scientific publications [31]. Such metadata are reasonably standardized among online news sources and they offer a headline-like summary of the article, typically designed for sharing on social media, making them suitable for topic detection and sentiment analysis. In total, we collected 26,077,939 articles from front pages of 172 online news sources (Table 1), with the full list of contributing sources in Table S1 in Multimedia Appendix 1.

Contemporary news sites rapidly change their content, which is spread through multiple sections, so it is difficult to gauge the level of attention received by particular articles. Front pages of news sites should be reliable reflections of the information many users are exposed to, since they are the main points of entry. This is opposed to other article collection strategies, such as RSS (Really Simple Syndication) or downloading the entire website content, that can provide limited control over assessing how many people have actually read any given article [16,32]. Focusing our efforts on articles from landing pages of major online news sources enabled us to assess the number of COVID-19–related articles that a large proportion of online news consumers were exposed to.

**Table 1:** Number of online news sources and collected articles per country.

Country	Online news sources (N=172), n (%)	Collected articles (N=26,077,939), n (%)
Canada	13 (7.5)	1,269,200 (4.8)
Australia	8 (4.6)	1,124,859 (4.3)
Italy	13 (7.5)	1,526,521 (5.8)
The United Kingdom	21 (12.2)	4,977,792 (19.0)
The United States	33 (19.1)	4,388,383 (16.8)
France	9 (5.2)	1,951,608 (7.4)
Germany	18 (10.4)	2,348,403 (9.0)
Ireland	8 (4.6)	905,598 (3.4)
International	6 (3.4)	462,989 (1.7)
New Zealand	5 (2.9)	651,050 (2.4)
Russia	19 (11.0)	3,348,825 (12.8)
Spain	19 (11.0)	3,122,711 (11.9)

### Topic Models

For each article we extracted, we analyzed the text content of the metadata title and description to determine whether the article could be associated with one of the following topics: cat, sport, Merkel, Putin, Johnson, Biden, Trump, cancer, climate, or COVID-19. Non–COVID-19 topics were selected to provide a reference with large expected volumes of coverage (ie, politicians) that cover a wide range of sentiments (еg, *cat* as nonnegative and *cancer* as negative). Each topic was identified on the basis of the keywords presented in Table 2. The only normalization we applied to the words for topic identification was case folding, otherwise the words were not stemmed nor lemmatized. Topics that were used solely for sentiment analysis—cat, sport, climate, and cancer—were not identified for non-English online news sources.

We chose keywords for each topic in order to maximize the precision of topic identification. Because we focused on titles and descriptions, mentions of specific keywords here made it unlikely that they were only tangentially relevant to the article at hand (eg, explicit mentions of politicians’ names). In the case of COVID-19, we tested the extent to which our topic detection misclassified the topic by identifying COVID-19 articles in the pre–COVID-19 era of 2015 to 2019. Out of 21,693,591 articles, only 7375 (0.03%) were erroneously identified as related to COVID-19, demonstrating high precision of the selected keywords. In the majority of cases, misclassifications stemmed from mentions of lockdowns, which were chiefly gun related, but in the case of the United Kingdom, they were even related to a seagull attack on a school. Subsequent subtopic identification stratified the different threads of COVID-19 coverage, providing a wider set of keywords.

**Table 2:** Keywords employed for topic detection.

Topic	Keywords by language							
	English	German	French	Spanish	Italian	Russian		
**COVID-19**								
	coronavirus	coronavirus	coronavirus	coronavirus	coronavirus	коронавирус		
	covid	covid	covid	covid	covid	covid ковид		
	lockdown	lockdown	lockdown couvre-feu	lockdown confinamiento	lockdown contenimento	lockdown локдаун		
	quarantine	quarantäne	quarantaine	cuarantena	quarantena	карантин		
	pandemic	pandemie	pandémie	pandemia	pandemia	пандемиа		
	N/A^a^	corona-	N/A	N/A	N/A	N/A		
Merkel	merkel	merkel	merkel	merkel	merkel	merkel меркел		
Trump	trump	trump	trump	trump	trump	trump трамп		
Biden	biden	biden	biden	biden	biden	biden байден		
Johnson	boris johnson	boris johnson	boris johnson	boris johnson	boris johnson	boris johnson борис джонсон		
Putin	putin	putin	putin	putin	putin	putin путин		
**Climate**								
	global warming	—^b^	—	—	—	—		
	climate change	—	—	—	—	—		
	climate crisis	—	—	—	—	—		
**Cat**								
	cat	—	—	—	—	—		
	kitten	—	—	—	—	—		
**Sport**								
	baseball	—	—	—	—	—		
	major league	—	—	—	—	—		
	champion's league	—	—	—	—	—		
	football	—	—	—	—	—		
	nfl	—	—	—	—	—		
	premier league	—	—	—	—	—		
	basketball	—	—	—	—	—		
	soccer	—	—	—	—	—		
	nba	—	—	—	—	—		
Cancer	cancer	—	—	—	—	—		

^a^N/A: not applicable; this keyword, which is specific to the German language because of its compound nature, was only found in German news sources.

^b^The topics *climate*, *cat*, *sport*, and *cancer* were not identified in non–English-language online news sources, as these were solely employed for sentiment analysis.

We further identified subtopics within COVID-19 news coverage by a similar keyword-based approach. Since many subtopic words can have several forms (eg, dead, died, and dies), we stemmed the words associated with each subtopic (eg, healthy and healthier are both stemmed to health); these are presented in Table 3. An article was identified as pertaining to a subtopic if, after stemming its title and description, a token corresponding to a stemmed keyword in Table 3 was identified.

**Table 3:** COVID-19 news subtopics.

Subtopic^a^	Stemmed keywords
Case	case
Crisis	crisi
Death	die, death
Disease	diseas
Distancing	distanc
Fear	fear
Health	health
Home	home
Hospital	hospit
Infection	infect
Isolation	isol
Lockdown	lockdown
Mask	mask
Outbreak	outbreak
Quarantine	quarantin
Spread	spread
Symptom	symptom
Test	test
Treatment	treatment
Vaccine	vaccin

^a^Each of the subtopics was identified by the stemmed keywords (ie, stemming).

### Sentiment Analysis of News Articles Using Vader

We used a well-established sentiment analysis tool, Vader [33], to identify emotionally polarized content, which has previously been applied to news media. It is suitable for short snippets of text, such as the titles and descriptions in our metadata. For a given piece of text, Vader provides a compound score between –1 and 1, with –1 being entirely negative, 0 being neutral, and 1 being entirely positive. For instance, the phrase “I find your lack of faith disturbing” offers a Vader score of –0.42, whereas the phrase “I find your lack of faith encouraging” gives a score of 0.5994. In our case, a sentiment score for a single article consists of a Vader compound score for the concatenation of the article title and description.

Novel topics are associated with many subject-specific keywords and phrases (eg “social distancing” and “lockdown” for COVID-19). Applying sentiment analysis to text with novel keywords can result in software being unable to correctly annotate polarization. We assessed Vader sentiment annotations on articles identified as one of the subtopics in Table 3. This revealed an artifact of the tool, wherein “positive coronavirus test” was labeled as emotionally polarized in the positive direction by virtue of the word “positive.” In order to mitigate the effect of this subject-specific misclassification, the words “positive” and “negative,” for symmetry, were removed from articles related to coronavirus testing prior to applying Vader annotation.

### Estimating Topic Polarization: Relative Sentiment Skew

We examined whether coverage of a given topic was more emotionally polarized than another topic by contrasting their sentiment distributions. Directly comparing topic sentiment distributions between different online news sources is not sound. Different online news sources can be more sensational and negative or toned-down and neutral, which gives radically different sentiment distributions. To address this issue, we calculated whether specific topic coverage was more negative, positive, or neutral relative to other articles within a particular online news source.

For each article “*a*” (ie, that article’s title and description metadata) in English-language online news sources, we calculated the Vader compound sentiment score *sent(a)*. For all 2020 articles and topics, in a given online news source, we calculated the mean of the Vader compound sentiment scores, denoted as *μ_ONS,TOPIC_* (online news source [ONS]; equation 1). As a reference statistic for the distribution of sentiment scores not relating to a topic, we calculated the mean Vader score of the articles from a given online news source that were not identified as a given topic, denoted as *μ_ONS,TOPIC_* (equation 2):

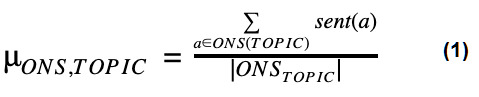

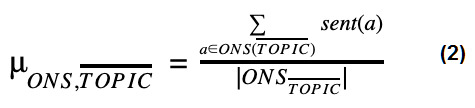





where *ONS* is a particular online news source, *TOPIC* is a topic from Table 2 or Table 3, *ONS(TOPIC)* is the set of articles on a given topic from a particular online news source, and |*ONS_TOPIC_*| is the total number of articles on a given topic in that online news source. A set of articles not identified as a given topic from a particular online news source is denoted as *ONS_TOPIC_*.

For each topic in each online news source, we calculated the relative sentiment skew (*rsskew_ONS,TOPIC_*) between topic mean sentiment and the mean of all other articles in the given online news source (equation 3).

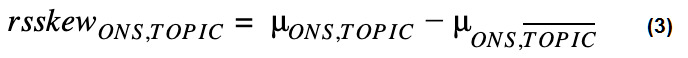



Relative sentiment skew is designed to indicate whether the sentiment score distribution of a particular topic is negatively or positively polarized, compared to other articles, for example, where a topic has one positive (score +1) and two negative articles (each with score −1), and there are seven other nontopic articles that are all positive (each with score +1). In this instance, the relative sentiment skew metric *rsskew_ONS,TOPIC_* is –1/3 – 7/7 = –1.33, which indicates greater negativity. Note, we do not account for sample size variation, as the denominator is generally very large (ie, in the thousands).

## Results

### One-Quarter of 2020 News Coverage Was Pandemic Related, Suggesting Information Overload

We estimated the extent of COVID-19 coverage in online news media by identifying articles relating to the pandemic and comparing this number to the total number of articles between January and October 2020.

For each online news source, we performed topic detection, categorizing each article title and description as relating to the coronavirus—topic called COVID-19—if the title and description contained any keyword of a specific set, given in Table 2. Keywords for this simplified topic detection model were chosen to maximize the precision and accuracy of content identification in order to avoid cross-contamination with other topics. In English, these keywords included *covid*, *coronavirus*, *lockdown*, *quarantine*, and *pandemic*, but not, for example, *hospital* and *death*. The keywords were adjusted for the six languages that we used in this study: English, German, French, Spanish, Italian, and Russian (Table 2).

**Figure 1:**
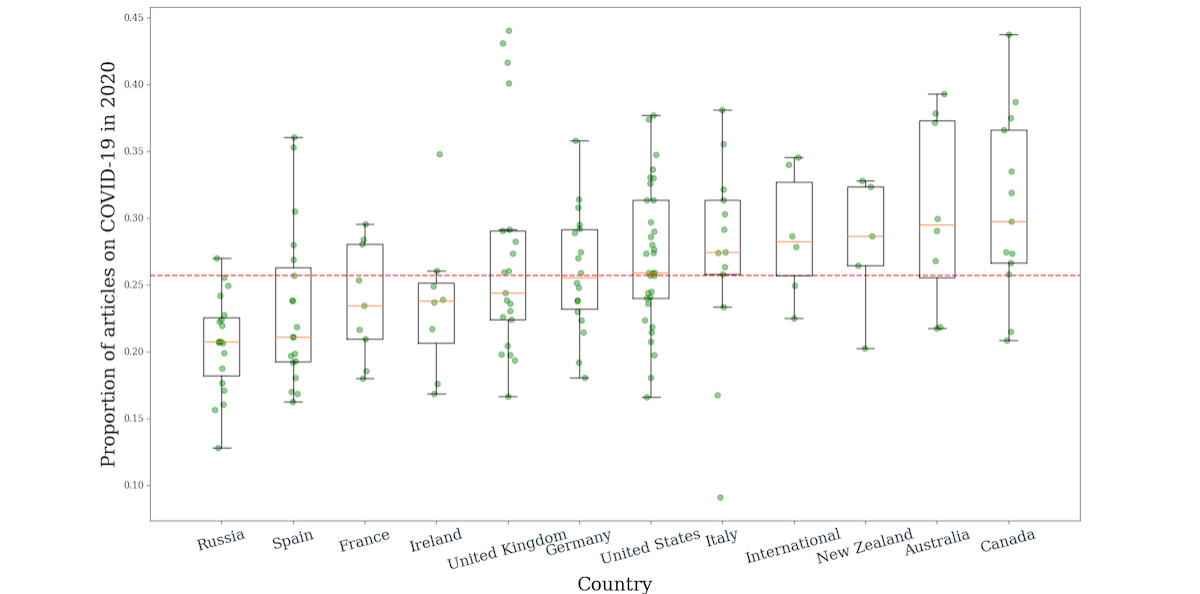
The extent of coronavirus coverage in 2020. We calculated the proportion of all COVID-19 articles as the proportion of all front-page articles. Proportions were calculated for each online news source separately and then aggregated at the national level. The green points represent the individual coverage of each online news source. The yellow line in each box represents the median; the upper and lower whiskers represent the 75th and 25th percentiles, respectively. The red dotted line indicates mean proportion across all online news sources.

COVID-19 featured in 25.3% of the news articles (1,135,561/4,477,867) across the online news sources (Figure 1). While this proportion varied between countries, it was consistently large, with the lower bound at 20% and the upper bound at 30%. Thus, even using our relatively simple topic detection model, we were able to demonstrate that 2020 online news coverage was dominated by COVID-19.

To provide a point of reference to coverage of COVID-19, we identified other global topics with regular and topical media presence (Table 2). We selected topics such as Donald Trump (Trump), Joe Biden (Biden), Boris Johnson (Johnson), Angela Merkel (Merkel), and Vladimir Putin (Putin). Unsurprisingly, mentions of politicians are most common in their home countries (Figures S1-S5 in Section 2 of Multimedia Appendix 1) (eg, Vladimir Putin in Russia). Nonetheless, each politician received an order of magnitude less media attention than COVID-19 in 2020. Specifically, mean coverage of COVID-19 across 11 countries was 25.66%, whereas politicians received 2.50% for Trump, 0.45% for Biden, 0.18% for Putin, 0.17% for Johnson, and 0.09% for Merkel. Higher coverage of COVID-19 was also reflected at the national level. Although 2020 was an election year in the United States, *Trump* received a mean of 15.29% of 2020 media mentions in US online news sources, as opposed to 25.91% of mean mentions for COVID-19. Furthermore, US articles in 2020 mentioning both Trump and COVID-19 accounted for a mean coverage of 3.82%.

To provide temporal perspective on the media attention of COVID-19, we plotted the proportions of global coverage in the period from January to October 2020 (Figure S6 in Section 2 of Multimedia Appendix 1). COVID-19 attention in 2020 spiked between March and May, coinciding with many countries following the Chinese strategy of lockdown and other distancing measures. Proportion coverage leading up to the second COVID-19 wave in Europe and the United States did not reach levels seen from March to May but did stay above 20% in the regions we considered. These results provide a quantification of the extent of media attention received by COVID-19 across countries and languages, reflecting the global and protracted impact of the pandemic.

### COVID-19 News Sentiment Analysis Suggests Heterogeneity of Coverage

The emotion that news coverage of COVID-19 evokes is an important factor in a society’s response to the pandemic [25]. We addressed this issue by sentiment analysis contrasting emotional polarization of COVID-19 news with that of certain reference topics and all non–COVID-19 articles for each online news source. Such contrast allows us to determine whether sentiment distribution of COVID-19 coverage was polarized compared to reference topics and other online news for a given online news source.

To quantify the emotional content of news article text, we employed Vader, which has been previously applied to news article analysis [33,34] (see Methods section). For each online news source whose primary language was English (91/172, 52.9%), we created Vader annotations for both the title and description of each of its articles. We grouped articles by their annotated topics (Table 2) to provide a comparison with COVID-19 sentiments. Politicians—Merkel, Trump, Johnson, Biden, and Putin—were used as reference points for subjects with frequent coverage. We selected four additional topics to offer intuitive reference points on the positive and negative sentiment spectrum: cat, sport, climate, and cancer. The topics *cat* and *sport* were used, as they would not necessarily be associated with negative sentiments. Likewise, the topics of *climate*—identified by the key phrases global warming, climate crisis, and climate change—and *cancer* were used as references that we would expect to be associated with negative emotions. Altogether, individual sentiment annotations for each online news source were grouped by one of the topics: cat, sport, Merkel, Johnson, Biden, Trump, COVID-19, and cancer.

**Figure 2:**
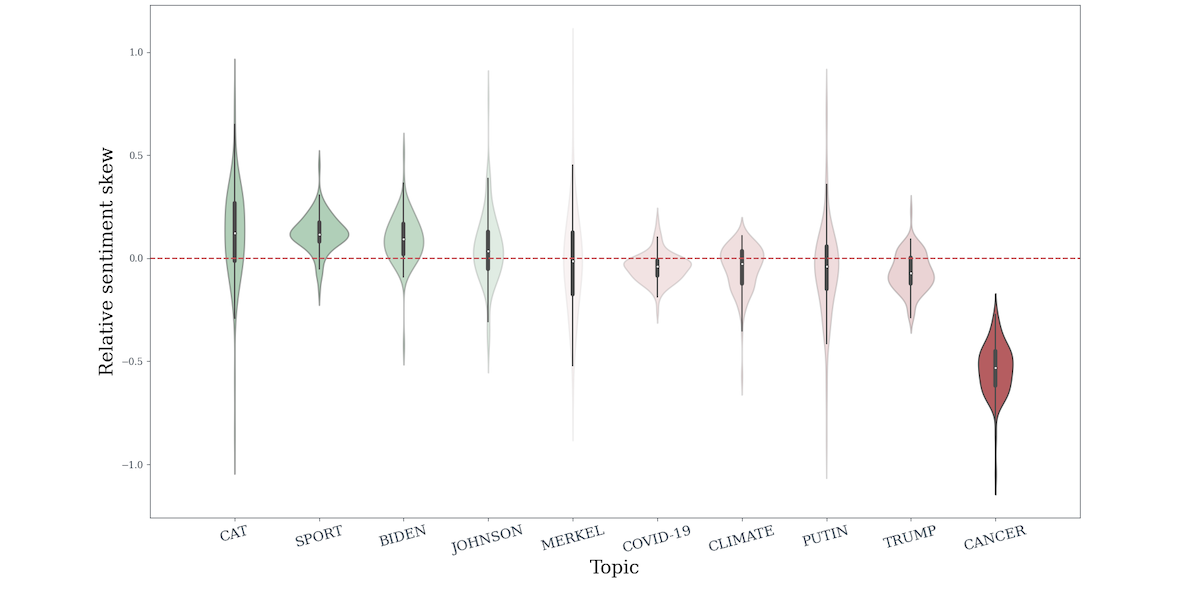
Relative sentiment skew (*rrskew*) of COVID-19 coverage. Each article title and description from each English-language online news source (ONS) received a Vader sentiment compound score between –1 and 1 (most negative and most positive, respectively). We noted the difference in mean sentiment for a specific topic and mean sentiment for other 2020 articles in a given online news source (*rsskew_ONS,TOPIC_*; see Methods section). The density of the relative sentiment skew is plotted for each topic. Distributions are colored green if their relative sentiment skew was predominantly positive or red if predominantly negative (Table 4). Intensity of the color is scaled by the distance from the red dotted line at 0, which indicates a lack of difference between topic sentiment and all other articles in a given online news source.

For each online news source and topic, we calculated the relative sentiment skew statistic (*rsskew_ONS,TOPIC_*; see Methods section), which measures the polarization of a given topic within an online news source. We noted how many topics had a negative or positive relative sentiment skew value (Table 4). For the nonpolitician non–COVID-19 topics (ie, cat, sport, and cancer), skew was in the expected direction, suggesting that they are appropriate references for assessing sentiment of COVID-19 articles. We noted negative relative sentiment skew values for 74 out of 91 (81%) English-language online news sources. Nonetheless, this observation cannot be taken as evidence of significant negative polarization, as these relative sentiment skew values are not substantially different than *rsskew*=0, which indicates no polarization. The mean relative sentiment skew for COVID was –0.04 (SD 0.07) (Table 4). Since cancer and COVID-19 are both diseases and exert a large burden on public health, one might expect their sentiment distributions from online news to be similar. However, COVID-19 sentiment distribution was not as extreme as that of cancer, which was 100% negative per online news source, and had a mean relative sentiment skew of –0.53 (SD 0.12). Perhaps surprisingly, the sentiment distribution for COVID-19 articles was more akin to coverage of climate, which was *a priori* expected to be negative, akin to cancer, or to subjects covering heterogenous topics by virtue of their wide-ranging implications for society, such as politicians (Figure 2). This, however, can be an indicator of topics being intertwined: since heads of state are responsible for the pandemic response, they can be expected to be mentioned in relation to COVID-19.

**Table 4:** English-language online news sources^a^, with positive (≥0) or negative (<0) relative sentiment skew of 2020 articles on a given topic.

Topic	Positive online news sources, n (%)	Negative online news sources, n (%)	Relative sentiment skew, mean (SD)	Total articles^b^, n
Cat (n=87)	64 (74)	23 (26)	0.12 (0.23)	2746
Sport (N=91)	84 (92)	7 (8)	0.12 (0.08)	63,155
Biden (n=90)	75 (83)	15 (17)	0.09 (0.11)	38,949
Johnson (n=90)	57 (63)	33 (37)	0.04 (0.17)	22,613
Merkel (n=79)	38 (48)	41 (52)	–0.01 (0.25)	2011
COVID-19 (N=91)	17 (19)	74 (81)	–0.04 (0.07)	589,701
Climate (N=91)	38 (42)	53 (58)	–0.04 (0.11)	7195
Putin (n=88)	33 (38)	55 (63)	–0.05 (0.23)	5179
Trump (N=91)	24 (26)	67 (74)	–0.06 (0.09)	157,702
Cancer (N=91)	0 (0)	91 (100)	–0.53 (0.12)	9548

^a^We had 91 English-language online news sources in total; however, in cases where it was impossible to identify a certain topic in a given source, it was left out.

^b^The total number of articles we identified as being associated with a given topic across all online news sources.

These results suggest that the sentiment of COVID-19 coverage in online news media is heterogeneous and is certainly not as clearly polarized as cancer, though the volume of coverage might play a role (Figure S7 in Section 3 in Multimedia Appendix 1). One explanation could be the all-permeating nature of the pandemic, where it becomes background to most reporting. Therefore, COVID-19 articles cannot all be categorized as fully negative, contrary to the expectation of pandemic subject matter. In fact, on average, they appear to not be polarized in either the positive or negative direction, especially when compared to reference topics. This suggests that coverage of COVID-19 was highly heterogeneous, with many themes contributing to the totality of messaging.

### Highly Sentiment-Negative Subtopics Account for 16% of COVID-19 Coverage, Suggesting Emotional Pressure

We studied the text content of COVID-19–related title and description metadata to reveal the leading themes associated with heterogeneous pandemic reporting.

We investigated article subtopics and calculated the most commonly used words and bigrams (ie, consecutive combinations of two words) to demonstrate the most frequent mentions in COVID-19 coverage. For each of the 91 English-language online news sources, we calculated the ranks of single words and bigrams in articles that pertained to COVID-19. The articles were further subdivided within each online news source as negative (Vader score <–0.2; 247,542 articles), positive (Vader score >0.2; 192,643 articles), or all (any Vader score; 589,709 articles). This subdivision aimed to reveal whether certain keywords or bigrams were more frequently associated with differently polarized text. For each subdivision, we averaged the individual online news source ranks of each word and bigram found across all 91 online news sources. In Table 5 we present the top 20 highest-ranking words and bigrams across all 91 online news sources. Words and bigrams in Table 5 reveal many themes that are intuitively associated with the coronavirus, such as testing, vaccine, death, etc. In particular, negative articles had unique top words and bigrams that are intuitively associated with negative emotions. In the singletons, these were *death*, *crisi*, and *fear*, while in bigrams these were *covid_crisi*, *covid_death*, *coronavirus_death*, and *death_toll*—note that words are stemmed.

**Figure 3:**
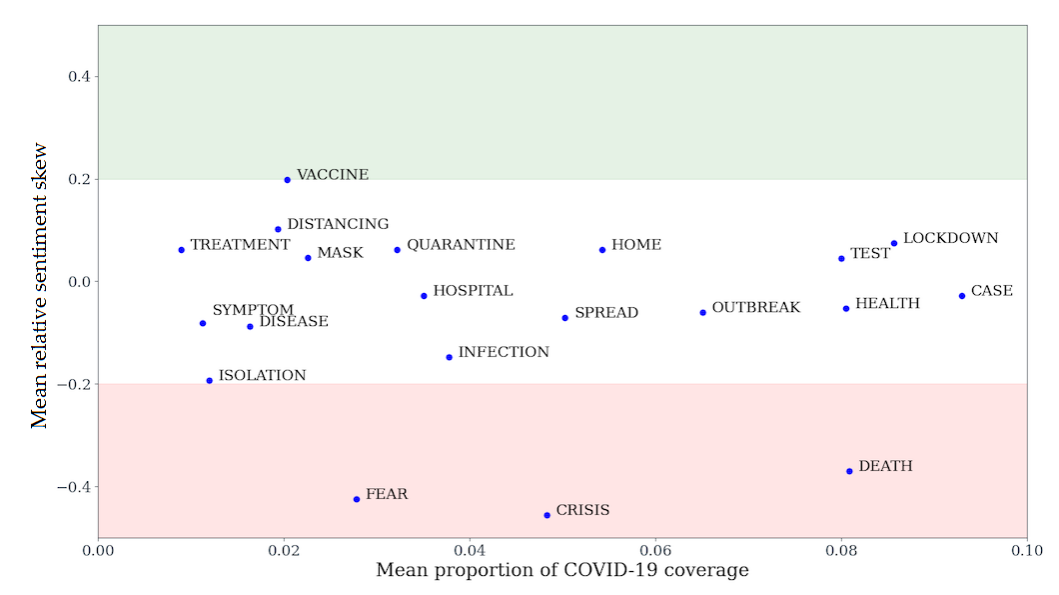
COVID-19 subtopic coverage and sentiment means. We calculated the mean coverage and mean sentiment of each subtopic. Coverage is expressed as the mean of ratios of subtopics in a given online news source against all COVID-19 articles in the same online news source. Sentiment is the mean of the subtopic relative sentiment skew for all online news sources. The shaded areas illustrate regions with relative sentiment skew above 0.2 (green), between 0.2 and –0.2 (white), and below –0.2 (green).

To calculate the news coverage proportion related to these top themes, we created a constrained set of COVID-19 subtopics, based on the highest-ranking words and bigrams from Table 5. We removed terms that pointed to nonspecific coronavirus coverage, such as *COVID*, *coronavirus*, *pandemic*, or *news*. We extended the list of subtopics to include those we did not find in Table 5 but considered as strongly related to COVID-19 coverage, such as *hospital*, *quarantine*, *symptom*, or *isolation*, with a full list of subtopics in Table 3. For each subtopic, we calculated the proportion of COVID-19 coverage per online news source (Figure S8 in Section 4 of Multimedia Appendix 1) and relative sentiment skew per online news source for the subtopic (Figure S9 in Section 4 of Multimedia Appendix 1). The means of coverage and sentiments per online news source are plotted against each other in Figure 3. The subtopics in Table 3 account for a mean of 67.14% of all COVID-19 articles across English-language online news sources. Of these, the top three are *case*, *lockdown*, and *death*, which account for a mean of 9.29%, 8.56%, and 8.08% of COVID-19 articles, respectively. Figure 3 and Figure S9 in Multimedia Appendix 1 suggest that out of *case*, *death*, and *lockdown*, only *death* carried a firmly polarized sentiment, with *case* and *lockdown* not being significantly skewed in either positive or negative directions. The words *fear*, *crisis*, and *death* unsurprisingly indicated substantial negative polarization (Figure 3).

We analyzed the impact on news sentiment in 2020 of the three most negative subtopics, *fear*, *crisis*, and *death* (Figure 3). For each online news source, we calculated the mean sentiment of all the 2020 articles after removing articles that mentioned one of those three topics. For all 91 online news sources, removing articles mentioning one of the top three negative topics resulted in a statistically significant—at the level of .05 with Bonferroni correction—shift toward mean positive sentiment (Section 5 in Multimedia Appendix 1). By contrast, removal of all the sentiment-heterogeneous (Figure 2) articles from all 2020 articles resulted in a significant shift toward mean positive sentiment in 40 out of 91 (44%) online news sources, a significant shift toward mean negative sentiment for 11 out of 91 (12%) online news sources, and no statistically significant result for 39 out of 91 (43%) online news sources. Altogether, articles mentioning *fear*, *crisis*, and *death* accounted for a mean of 16% of COVID-19 articles across 91 online news sources; due to their highly polarizing nature, they may play a significant role in shaping societal perception of the pandemic.

Of the three most negative topics, *fear*, *crisis*, or *death*, the latter was the most frequently mentioned for COVID-19 (Figure 3). In total, *death* was mentioned in the context of COVID-19 in 2.33% of all coverage in 91 English-language online news sources. All *death* mentions in 2020 in 91 English-language online news sources accounted for a mean coverage of 5.74%, whereas in the pre–COVID-19 period of 2015 to 2019, they accounted for 4.07%. Therefore, we can identify that *death* in the context of COVID-19 constituted a significant proportion of negatively associated coverage that appears to have contributed to overall death reporting in the news.

These results demonstrate that even though the overall coverage of COVID-19 in 2020 was not significantly polarized by sentiment, there was a nontrivial proportion of negative news that contributed to overall reporting negativity in 2020.

**Table 5:** Top words and bigrams in English-language countries.

Rank		Words and bigrams by polarization^a^			
		Negative	All	Positive	
**Single words**					
	1	coronavirus	coronavirus	coronavirus	
	2	covid	covid	covid	
	3	pandem	pandem	pandem	
	4	new	new	new	
	5	peopl	peopl	help^b^	
	6	say	case	say	
	7	crisi^b^	say	peopl	
	8	health	health	test	
	9	case	test	health	
	10	death^b^	outbreak	case	
	11	outbreak	week	us	
	12	virus	us	home	
	13	test	virus	week	
	14	could	could	one	
	15	govern	day	time	
	16	us	one	day	
	17	countri	govern	govern	
	18	one	home	could	
	19	week	countri	work^b^	
	20	fear^b^	time	outbreak	
**Bigrams**					
	1	coronavirus_pandem	coronavirus_pandem	case_covid	
	2	coronavirus_crisi	case_coronavirus	coronavirus_pandem	
	3	coronavirus_outbreak	case_covid	posit_test	
	4	health_public	coronavirus_spread	health_public	
	5	posit_test	distanc_social	coronavirus_outbreak	
	6	case_coronavirus	health_public	case_coronavirus	
	7	coronavirus_spread	covid_test	covid_pandem	
	8	coronavirus_new	coronavirus_outbreak	distanc_social	
	9	case_covid	covid_pandem	coronavirus_lockdown^b^	
	10	distanc_social	coronavirus_new	coronavirus_spread	
	11	covid_crisi^b^	coronavirus_crisi	covid_test	
	12	covid_test	case_new^b^	covid_vaccin^b^	
	13	covid_pandem	covid_outbreak	home_stay	
	14	coronavirus_due^b^	posit_test	coronavirus_test^b^	
	15	covid_death^b^	minist_prime	covid_posit^b^	
	16	second_wave^b^	home_stay	minist_prime	
	17	death_toll^b^	first_time^b^	like_look^b^	
	18	coronavirus_death^b^	around_world^b^	covid_outbreak	
	19	amid_coronavirus^b^	covid_spread^b^	coronavirus_new	
	20	two_week	two_week	coronavirus_vaccin^b^	

^a^For each of the 91 English-language online news sources, we calculated the most common words and bigrams and grouped these by Vader scores: >0.2 for positive, <–0.2 for negative, and any score for all. We averaged the ranks of words and bigrams across all online news sources, and here we present the top 20 for each subdivision. The words in the table are stemmed.

^b^These entries indicate elements that can be found in the top 20, only in the specific subdivisions of *positive*, *all*, or *negative*.

## Discussion

In this work, we compiled the largest data set on COVID-19 news, collating over 26 million articles from the front pages of 172 major online news sources from 11 countries. We have made this database publicly available at SciRide [28]. We first investigated trends in COVID-19–related news with respect to all front-page articles in 2020. We next used sentiment analysis to determine whether COVID-19 coverage was more polarized than other topics. Finally, we analyzed the leading subtopics in COVID-19 coverage and assessed their sentiment polarization. We demonstrated that 25% of front-page articles in traditional news media between January and October 2020 concerned COVID-19. Sentiment analysis demonstrated that pandemic coverage cannot be simply categorized as negatively polarized by virtue of disease association, pointing to heterogenous reporting. However, there was an increased incidence in reporting of negatively associated topics, in particular concerning *death*. Our results provide a data-driven foundation on policy communication surrounding distancing measures.

NPIs are drastic measures that reduce casualties before vaccinations and/or treatment regimens become widespread. Such methods, however, are only effective with societal adherence. Information received by a population in times of a pandemic shapes collective adherence to policies introduced to stem its spread. Currently, the internet is the primary source of health information for people in developed countries [35].

Comprehensive analysis of COVID-19 information received by a population would require thorough analysis of all possible internet news sources and all users’ exposure to each piece of information received. The online ecosystem is extremely heterogeneous, with channels of information discovery spread across traditional news sites, blogs, social media, and many others. Within each of such platforms, information itself can take different forms (eg, text length and format). How users interact with the information also has a great effect on the amount of attention a given piece of information receives and the degree of influence it has (eg, extent of sharing on social media or a more visible position on a website). Analysis of COVID-19 information from all online sources is not tractable.

Direct access to major news sites accounts for 76% of media consumption online [29]. The landing pages of such outlets implicitly capture articles that might have been seen by online users. Thus, our analysis of content from front pages of major news sites should encompass a significant proportion of sources shaping knowledge of the pandemic covering reporting across different languages and geographies. In total, we curated a data set of 26 million articles from 172 major web traffic–generating online news sources in 11 countries.

We identified COVID-19–relevant articles as well as a selection of other topics to serve as reference points for both coverage volume and sentiment analysis. As a standardized common denominator among articles, we analyzed the metadata titles and descriptions, where the main subject matter can be expected to be referenced. We employed a facile topic identification method using a limited set of keyword mentions. We chose a limited number of keywords to make it unlikely that an article would not make the corresponding topic its subject matter if it were referenced in its metadata title and description (eg, politician’s name). This avoided the caveat of tangential references to certain topics mentioned in the full article body or ambiguities that might arise by using more sophisticated topic modeling algorithms [36]. Unlike more complex topic modeling methods, or even using a wider set of keywords, our approach did not capture much more subtle references to these topics, and so we will have underestimated total coverage.

Nonetheless, even using our simple approach, we still identified a nontrivial number of COVID-19 articles on the front pages of our online news sources. We estimate that a mean of 25% of our sample of front-page articles from 11 countries in 2020 mentioned COVID-19 in their titles and descriptions. Our method had reduced topic identification recall by not accounting for more subtle references to COVID-19, and the totality of the articles was certainly contaminated by retrieval of erroneous links that were not actual news articles. Therefore, the actual proportion of articles on the front pages of news sources referencing COVID-19 might have indeed been higher. We envision that the amount of reporting on a topic of general interest like COVID-19 needs to be balanced. Too little information might leave the population underinformed and ill-equipped to respond appropriately. Too much coverage risks obscuring information that is crucial for individuals to understand the pandemic and how to stay safe.

Reporting on the pandemic could have wider implications than only its basic informative function. It is unknown what effect regular reporting on cases, casualties, and containment methods could have on adherence to distancing rules or mental health [19,37-40]. Though current sentiment analysis methods fall short of identifying complex nuance, they offer a good approximation for the position of text on the emotional spectrum (ie, negative, neutral, or positive). By employing sentiment analysis, we found that overall COVID-19 reporting was not markedly polarized in either a positive or negative direction, as opposed to cancer. It is contrary to what might be expected by virtue of the pandemic subject matter, suggesting heterogeneity in reporting. Such heterogeneity might be due to the sheer scale of the pandemic, the consequences of which have permeated much of everyday life. Nevertheless, we found that negatively polarized COVID-19 articles mentioning death, fear, and crisis accounted for 16% of pandemic articles, with death being most widely referenced. Such a nontrivial volume of negatively associated articles significantly skews the sentiment of 2020 reporting toward the negative direction.

Our results offer a quantification of COVID-19 reporting that substantiates widespread qualitative observations (eg, the pandemic received an unprecedented amount of media attention). Our analysis offers insights for shaping discussion on health communication in order to maximize the effect of control policies. Our retrospective analysis of health communication during the first two waves indicates signs of information and emotional overload that might have obscured understanding of policy. We hope that our findings will inform how best to communicate so as to minimize the risk of subsequent waves while vaccination regimens are introduced.

## Appendix

Multimedia Appendix 1 Supplementary materials.

## Abbreviations

GDELT: Global Database of Events, Language, and Tone
NIHR: National Institute for Health Research
NPI: nonpharmaceutical intervention
ONS: online news source
RSS: Really Simple Syndication
*rsskew*: relative sentiment skew
