# Correction: Quantifying Online News Media Coverage of the COVID-19 Pandemic: Text Mining Study and Resource

**DOI:** 10.2196/31544

**Published:** 2021-07-14

**Authors:** Konrad Krawczyk, Tadeusz Chelkowski, Daniel J Laydon, Swapnil Mishra, Denise Xifara, Benjamin Gibert, Seth Flaxman, Thomas Mellan, Veit Schwämmle, Richard Röttger, Johannes T Hadsund, Samir Bhatt

**Affiliations:** 1 Department of Mathematics and Computer Science University of Southern Denmark Odense Denmark; 2 Department of Management in the Network Society Kozminski University Warsaw Poland; 3 Department of Infectious Disease Epidemiology MRC Centre for Global Infectious Disease Analysis Imperial College London London United Kingdom; 4 Nupinion London United Kingdom; 5 Department of Mathematics Imperial College London London United Kingdom; 6 Department of Biochemistry and Molecular Biology University of Southern Denmark Odense Denmark; 7 Section of Epidemiology Department of Public Health University of Copenhagen Copenhagen Denmark

In “Quantifying Online News Media Coverage of the COVID-19 Pandemic: Text Mining Study and Resource” (J Med Internet Res 2021;23(6):e28253), one error was noted.

Due to a system error, the name of one author, Benjamin Gibert, was replaced with the name of another author on the paper, Seth Flaxman. In the originally published paper, the order of authors was listed as follows:

Konrad Krawczyk, Tadeusz Chelkowski, Daniel J Laydon, Swapnil Mishra, Denise Xifara, Seth Flaxman, Seth Flaxman, Thomas Mellan, Veit Schwämmle, Richard Röttger, Johannes T Hadsund, Samir Bhatt

This has been corrected to:

Konrad Krawczyk, Tadeusz Chelkowski, Daniel J Laydon, Swapnil Mishra, Denise Xifara, Benjamin Gibert, Seth Flaxman, Thomas Mellan, Veit Schwämmle, Richard Röttger, Johannes T Hadsund, Samir Bhatt

In the originally published paper, the ORCID of author Benjamin Gibert was incorrectly published as follows:

0000-0002-2477-4217

This has been corrected to:

0000-0001-8457-3137

The correction will appear in the online version of the paper on the JMIR Publications website on July 14, 2021, together with the publication of this correction notice. Because this was made after submission to PubMed, PubMed Central, and other full-text repositories, the corrected article has also been resubmitted to those repositories.

